# Exploration of Alkyne-Based Multilayered 3D Polymers and Oligomers: Subtle Aggregation-Induced Emission, Chromium(VI) Ion Detection, and Chiral Properties Characterization

**DOI:** 10.3390/molecules29235641

**Published:** 2024-11-28

**Authors:** Sai Zhang, Qingzheng Xu, Xiuyuan Qin, Jialing Mao, Yue Zhang, Guigen Li

**Affiliations:** 1 School of Pharmacy, Changzhou University, Changzhou 213164, China; 2 School of Chemistry and Chemical Engineering, Nanjing University, Nanjing 210093, China; 3 School of Life and Science, Nanjing Normal University, Nanjing 210046, China; 4 School of Environmental Science and Engineering, Changzhou University, Changzhou 213164, China; 5 Department of Chemistry and Biochemistry, Texas Tech University, Lubbock, TX 79409-1061, USA

**Keywords:** multilayered 3D polymer, aggregation-induced emission, chromium (VI) ion detection, chiral properties characterization

## Abstract

This study investigates the synthesis and characterization of alkyne-based multilayered three-dimensional (3D) polymers, which exhibit a subtle aggregation-induced emission (AIE) phenomenon. The polymers demonstrate significant potential as fluorescent probes for the selective detection of chromium (VI) ions (Cr^6^⁺), showcasing their utility in environmental sensing applications. Additionally, the circular dichroism (CD) spectra reveal a pronounced cotton effect, indicative of chiral properties, while scanning electron microscopy (SEM) and dynamic light scatting (DLS) analysis reveal a distinctive rock-like surface morphology and Cr^6+^ sensitive anti-aggregation. These findings highlight the multifunctional capabilities of alkyne-based multilayered 3D polymers, suggesting their applicability in both fluorescence-based sensing and materials science. The insights gained from this research contribute to the development of advanced materials with tailored optical properties for environmental monitoring and other practical applications.

## 1. Introduction

**Figure 1:**
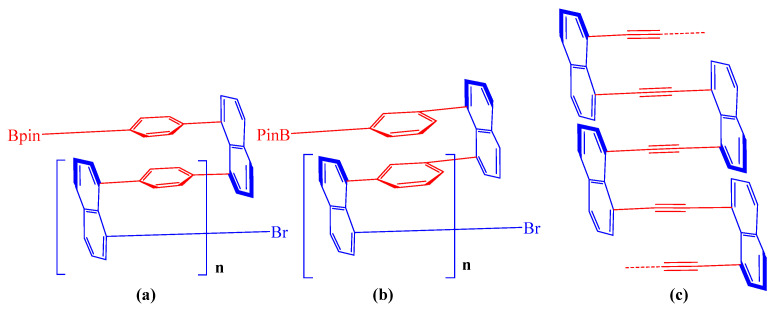
(**a**,**b**) Former synthesized multilayered 3D polymer. (**c**) This work.

Multilayer 3D chirality is an emerging area of research that focuses on the chirality of complex three-dimensional structures composed of multiple layers [1,2,3,4,5,6]. In natural systems, naturally occurring chiral structures can be found in biological macromolecules like proteins and DNA, where their specific three-dimensional arrangements are critical for biological function [7,8,9]. Protein folding structures frequently exhibit multilayer chirality as well [10]. The study of these structures informs the design of synthetic multilayered chiral materials [11,12,13,14,15]. In multilayered systems, layers of different materials or polymeric compounds are stacked to achieve unique properties [1,2,3,4]. Each layer can contribute distinct characteristics, such as optical, mechanical, or electrical properties, which can be tuned through precise fabrication techniques. In synthetic technology, the fabrication of multilayer 3D chiral architecture employs various advanced methods, including layer-by-layer Assembly and self-assembly [11,16] (Figure 1a,b). Layer-by-layer assembly is a technique that facilitates the alternating deposition of materials, allowing for precise control over both the composition and thickness of each layer. Self-assembly, on the other hand, leverages natural molecular interactions to spontaneously form ordered structures.

Multilayered 3D chiral structures exhibit unique physical phenomena, including optical activity [11,12,13,14,15,16], mechanical properties [16], and electronic behaviors [12]. Optical activity could enhance circular dichroism and selective light absorption characteristics [12], which are valuable for applications in optics and photonics. Mechanical properties improved strength, flexibility, and resilience due to the layered architecture, making these materials suitable for various structural applications [16]. For electronic behaviors, Certain multilayered chiral materials demonstrate novel electronic properties, such as chiral-induced spin selectivity, which has implications for spintronics and quantum computing [11].

With chiral auxiliaries and catalysts regulating stereochemistry, we have recently created a more pervasive C–C bonding bridge [12,13,14,15]. When exposed to UV light, the chiral multilayer 3D compounds aggregated and diluted displayed fascinating fluorescence. Polymers with a structurally compressed triple-columned/multilayered structure and related oligomers were synthesized. We also performed a computational evaluation of these targets, analyzing their aggregation-induced emission (AIE) features [11]. We created multilayered, chiral, triple-column 3D folding polymers in 2021, stacking at least seven layers using a variety of aromatic bridges.

In this work, with the use of various monomers and Sonagashira cross-coupling [17,18,19], we would like to present the design and synthesis of new achiral and chiral multilayer polymers in this study (Figure 1c). Furthermore, we provided details about their physical characteristics utilizing CD spectroscopy, SEM spectroscopy, aggregation-induced emission (AIE) [20,21,22], photoluminescence (PL), and UV-vis for discovering potential applications [21,22].

## 2. Experimental Section

### 2.1. Materials and Instrumentation

All procedures were magnetically stirred with anhydrous solvents in oven-dried glassware under Ar. Adding solvents, liquids, and solutions with syringes, stainless steel or polyethylene cannulas, rubber septa, or a weak Ar counter-flow. In Dewar containers, cooling baths of ice/water (0 °C) or dry ice/acetone (−78 °C) were generated. Heated oil baths were employed in high-temperature procedures. The solvents were removed using rotavapors at 40–65 °C. All yields are chromatographic and NMR yields separately.

Without further purification, all commercially available chemicals were used as received. Solvents such as CH_3_OH, toluene, EA, ether, DCM, dioxane, and acetone were used without additional purification. THF and DCM are delivered through an Innovation Technology solvent system.

The ^1^H NMR spectra were recorded on 400 MHz devices with TMS as an internal standard. The 1H NMR spectra were referenced using the residual solvent signal (=7.26 for CDCl_3_). The solvent signal was used in the ^13^C NMR spectra (=7.16 for CDCl_3_ and). Chemical changes in TMS were reported in parts per million (ppm). The data are described using chemical shift, multiplicity (singlet, doublet, triplet, multiplet), coupling constant (J, Hz), and integration. GPC data were collected using the TOSOH EcoSEC HLC-8420 GPC (Tokyo, Japan), which incorporates a dual-flow refractive index detector. In addition to the RI detector, a UV detector is included for use with UV–visible polymers. The installed columns have a range of 500–107 Da. For 25 min, samples were collected at a flow rate of 0.7 mL/min. For calibration, we employed polystyrene (PS) standards in our studies. Shanghai Lengguang F98 and Shanghai Lengguang 759s (Shanghai, China) were used to obtain photoluminescence spectra and UV–vis absorption. Sizes were measured by a nano laser particle-sized analyzer (Winner 802, Jinan Winner Particle Instrument Stock Co., Ltd., Jinan, China) respectively; CD spectroscopy was measured by JASCO J-1500 (Hachioji, Japan).

### 2.2. Synthetic Method of Monomer **1A**

1,8-dibromonaphtalene 1 (2 mmol) was added to a dry flask in an argon atmosphere. Et_3_N (6 mmol), PdCl_2_(PPh_3_)_2_ (0.04 mmol), CuI (0.1 mmol), and THF were then progressively added (20 mL). Then, 6 mmol of trimethylsilylacetylene was gradually added. The mixture was stirred at 90 °C in the oil bath for 12 h. Under reduced pressure, the solvent was removed, and the residue was left behind after being purified by chromatography on silica gel (PE), afforded the coupling product 1,8-dibromonaphtalene **1**.

**Figure 2:**
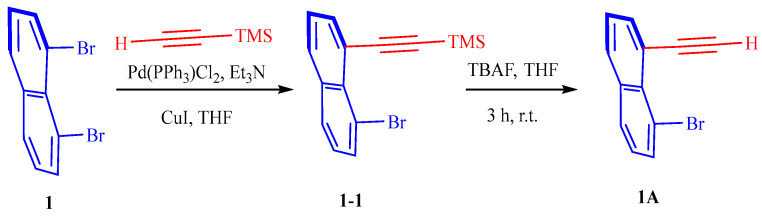
Synthetic method of monomer **1-1** and **1A**.

TBAF in THF (1.0 M, 0.9 eq) was added dropwise at 0 °C in the air to a solution of 1 (1.5 mmol) in THF (10 mL). The mixture was then swirled for 1 h. After applying sat. aq. NH_4_Cl to the reaction mixture, extraction with DCM was performed. The mixed organic layer was concentrated under a vacuum, dried over Mg_2_SO_4_, and washed with brine. By using column chromatography on silica gel (PE), the crude product was separated to produce **1A** (50% yield). 1-bromo-8-ethynylnaphthalene (**1A**): Brown solid, 50%, ^1^H NMR (400 MHz, CHLOROFORM-D) δ 7.92–7.70 (m, 4H), 7.40 (ddd, *J* = 8.2, 3.2, 1.6 Hz, ^1^H), 7.29–7.20 (m, 1H), 3.70–3.47 (s, 1H). MS(ESI): *m*/*z*, 231.15 [M + H]^+^ (Figure 2).

### 2.3. Synthetic Method of Polymer **2A**

**Figure 3:**
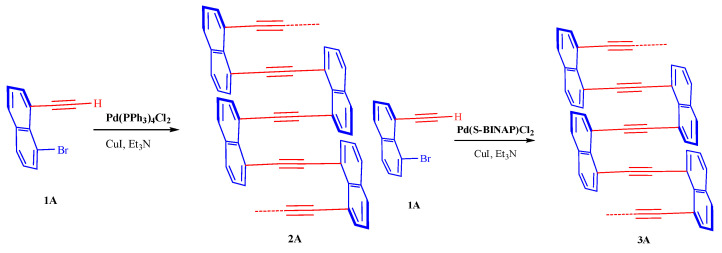
Synthetic method of polymer **2A** and **3A**.

A dry flask under an argon atmosphere containing **1A** was given successive additions of PdCl_2_(PPh_3_)_2_ (0.002 mmol), CuI (0.005 mmol), and Et_3_N (5 mL). The mixture was stirred for 12 h at 85 °C in the oil bath. After cooling to room temperature and removal of the solvent under decreased pressure, residue was obtained. This residue was washed with methanol and HCl at a concentration of 6.0 M to produce product **2A**: Brown solid, 85%, M_n_ = 49,199, M_w_ = 120,714, PDI = 2.454. ^1^H NMR (400 MHz, CHLOROFORM-D) δ 8.39–6.89 (m, Ar-H) (Figure 3).

### 2.4. Synthetic Method of Polymer **3A**

A dry flask under an argon atmosphere containing **1A** was given successive additions of Pd[S-BINAP]Cl_2_ (0.005 mmol), CuI (0.005 mmol), and Et_3_N (5 mL). The mixture was stirred for 12 h at 85 °C in the oil bath. After cooling to room temperature and removal of the solvent under decreased pressure, the mixture was chilled. In a single pot, the resultant mixture was then added to MeOH/HCl. The components that had precipitated were collected by filtration through a Buchner funnel and repeatedly rinsed with methanol and water. The solid was further dried to produce **3A**. Brown solid, 78%, M_n_ = 75,301, M_w_ = 98,069, PDI = 1.302. ^1^H NMR (400 MHz, CHLOROFORM-D) δ 7.74–6.84 (m, Ar-H).

## 3. Result and Discussion

### 3.1. Characteristics of UV-Vis Absorption

**Figure 4:**
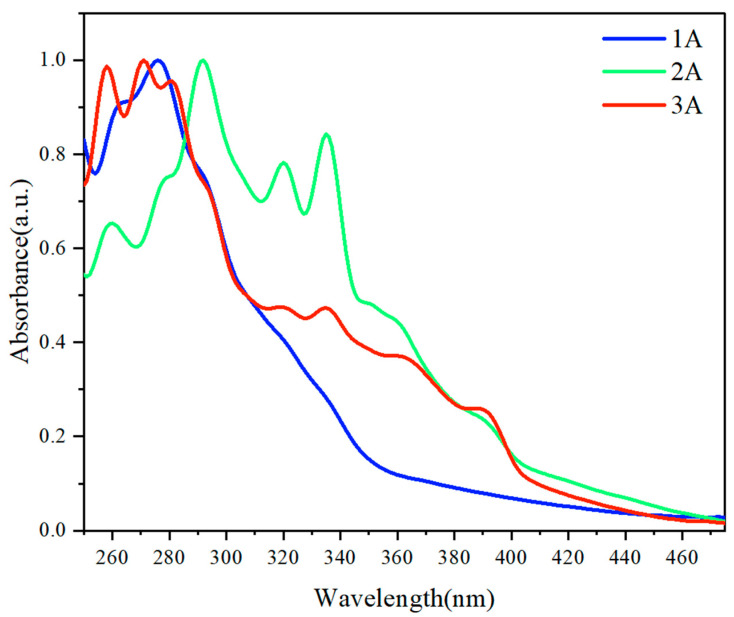
UV-vis absorption spectrum of **1A**, **2A**, and **3A**. Concentration: 0.05 mg/mL in THF.

In this research, we synthesized chiral and achiral multilayer 3D polymers based on alkyne and naphthalene substructures, with a particular emphasis on their UV-vis spectroscopic properties (Figure 4). The monomer 1-bromo-8-ethynylnaphthalene **1A** exhibits a prominent absorption peak within the range of 250 nm to 350 nm, with the highest intensity peak observed at approximately 275 nm. This peak is indicative of π-π* transitions characteristic of the naphthalene chromophore.

Upon polymerization, both chiral and achiral polymers (**2A** and **3A**) exhibited a broadened absorption spectrum extending beyond 250 nm, accompanied by a pronounced tailing phenomenon. Notably, the achiral polymer displayed its first and second-highest absorption peaks at approximately 295 nm and 340 nm, respectively. This deviation from the monomer’s absorption profile indicates significant alterations in the electronic environment and intermolecular interactions following polymer formation. The emergence of these new peaks can be attributed to the aggregation of polymer chains, which enhances electronic coupling and facilitates π-stacking interactions. Such interactions can lead to shifts in the absorption maxima, reflecting the delocalization of π-electrons across multiple polymeric units. The chiral polymer **3A** exhibits a distinct absorption peak at approximately 260 nm and 275 nm, which closely mirrors the absorption characteristics of the corresponding monomer. This finding suggests that, despite the structural modifications that occur during the polymerization process, the electronic transitions associated with the naphthalene moiety are largely preserved. The presence of optical activity in the polymer, as indicated in Figure 3 can be attributed to the utilization of an optically active catalyst during its synthesis, which imparts chirality to the resulting polymeric structure. Furthermore, the shifts in the absorption maxima of polymeric materials may be correlated with the extension of π-electron conjugation, which is noteworthy. Such shifts typically indicate alterations in the electronic environment of the chromophores, which can arise from increased delocalization of π-electrons across the polymer backbone.

The pronounced tailing phenomenon of **2A** and **3A** observed in both polymer systems suggests a potential aggregation-induced emission (AIE) effect. In the context of the polymers, the restriction of molecular motion due to intermolecular interactions may suppress non-radiative decay pathways, thus enhancing the fluorescence efficiency compared to the monomer. The differences in the spectral profiles between the chiral and achiral polymers further highlight the impact of chirality on the optical properties of the materials. The distinct absorption features of the achiral polymer suggest that the polymerization process leads to unique electronic environments that differ from those of the monomer and the chiral counterpart. The UV-vis spectroscopic analysis reveals significant changes in the absorption characteristics of the synthesized chiral and achiral polymers compared to the monomer. These changes underscore the influence of polymer structure on electronic transitions and highlight the potential for tailoring optical properties through strategic design in polymer synthesis.

### 3.2. Characteristics of Photoluminescence

To better assess the potential for aggregation-induced emission (AIE) and aggregation-induced enhanced emission (AIEE) phenomena, we employ photoluminescence to investigate the unique characteristics of a new alkyne multilayered 3D polymer in both its achiral and chiral forms. In AIE systems, the emission intensity typically increases as molecules aggregate. Photoluminescence measurements enable researchers to directly observe these changes in emission intensity under various conditions, such as concentration, solvent, or temperature. AIEE specifically focuses on the enhancement of emission intensity that occurs upon aggregation. By measuring photoluminescence, we can quantify this enhancement and confirm that aggregation leads to increased luminescence.

Two types of synthetic multilayer 3D polymers, **2A** and **3A**—chiral and achiral—are based on alkyne and naphthalene substructures, with a focus on their fluorescence characteristics in a solvent system comprising tetrahydrofuran (THF) and methanol. The solvent mixture was selected for its ability to elucidate the aggregation-induced emission (AIE) phenomena of the synthesized polymers.

**Figure 5:**
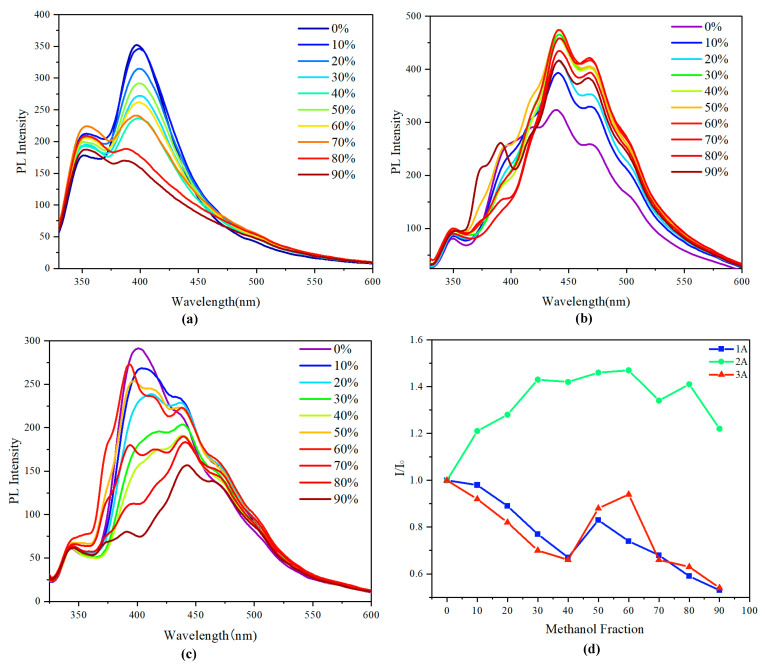
(**a**–**c**) PL spectra of **1A**, **2A**, and **3A** in THF/water mixtures with different methanol fractions (*f*_w_); *c* = 0.05 mg/mL; inset: fluorescence photographs of **1A**, **2A** and **3A** in THF/methanol system; Excitation wavelength: 317 nm. (**d**) Stern–Volmer plots of I_0_/I vs. methanol fraction of **1A**, **2A**, and **3A**.

The fluorescence behavior of monomer **1A** was initially investigated in the THF/methanol solvent system. The monomer exhibited no significant AIE phenomenon across the entire range of methanol fractions studied (Figure 5a). However, a subtle AIE effect was observed when the methanol fraction was between 40% and 50% (Figure 5d). In this specific range, there was a slight increase in fluorescence intensity, suggesting that some degree of aggregation may have occurred. Beyond this range, as the methanol fraction increased, the fluorescence intensity was consistently quenched. This quenching can be attributed to the increasing polarity of the solvent mixture, which likely disrupts the π-π stacking interactions and leads to enhanced non-radiative decay pathways, thus diminishing the emission efficiency.

In contrast, the achiral multilayer 3D polymer exhibited a pronounced AIE phenomenon, particularly within the methanol fraction range of 0% to 60% (Figure 5b,d). In this range, the fluorescence intensity increased significantly, indicating that the polymer chains were undergoing aggregation, which effectively restricted non-radiative decay pathways. Notably, even at a methanol fraction as high as 90%, the fluorescence intensity of the achiral polymer remained elevated compared to the 0% methanol fraction. This observation suggests that the polymer maintains a favorable conformation that promotes enhanced emission, likely due to effective intermolecular interactions and stabilization of the excited states through aggregation.

The behavior of the chiral multilayer 3D polymer closely resembled that of the monomer. A modest increase in fluorescence intensity was observed when the methanol fraction was between 40% and 60%, indicative of a limited AIE effect (Figure 5c,d). However, this increase was not as pronounced as that observed in the achiral polymer, and the overall fluorescence characteristics of the chiral polymer did not exhibit the same level of enhancement associated with aggregation. This suggests that the chiral architecture could impose specific conformational constraints that limit the degree of aggregation or alter the electronic interactions in such a way that the AIE effect is less favorable.

While the rigid backbone restricts intramolecular motions, which typically quench fluorescence in conventional systems, the presence of multiple layers and the overall three-dimensional architecture can enhance intermolecular interactions. These interactions may facilitate the formation of aggregation states that promote radiative transitions. Moreover, the subtle AIE effect may stem from the specific arrangement of chromophores within the polymer matrix. In the aggregated state, the close proximity of the chromophores can lead to enhanced π-π stacking interactions, thereby facilitating effective energy transfer and promoting emission. Additionally, the rigid structure may minimize non-radiative decay pathways, further contributing to the AIE phenomenon.

The differences in AIE behavior between the chiral and achiral polymers can be attributed to the inherent structural characteristics and intermolecular interactions facilitated by chirality. The achiral polymer’s ability to undergo significant aggregation and maintain enhanced fluorescence in a polar solvent environment could be due to more favorable packing arrangements and π-stacking interactions, which are less disrupted by the solvent. Conversely, the chiral polymer’s structural constraints could hinder effective aggregation, thus limiting the AIE phenomenon. Moreover, the solvent system plays a crucial role in modulating the fluorescence characteristics of these materials. THF, being a non-polar solvent, allows for better solvation of the polymer chains, while the addition of methanol introduces polarity that can influence the aggregation behavior. The balance between solvent polarity and the intrinsic properties of the polymers is vital in determining the extent of AIE observed. The fluorescence studies conducted in the THF/methanol solvent system reveal significant insights into the AIE phenomena of the synthesized chiral and achiral multilayer 3D polymers. While the monomer displayed limited AIE characteristics, the achiral polymer exhibited a robust AIE effect, underscoring the importance of polymer architecture in enhancing emission efficiency through aggregation. The chiral polymer, while showing some increase in fluorescence intensity, did not demonstrate a strong AIE effect, suggesting that chirality probably imposes constraints on the aggregation behavior. These findings highlight the potential for designing polymeric materials with tailored optical properties by manipulating structural and environmental factors, paving the way for future applications in optoelectronics, sensors, and advanced display technologies.

**Figure 6:**
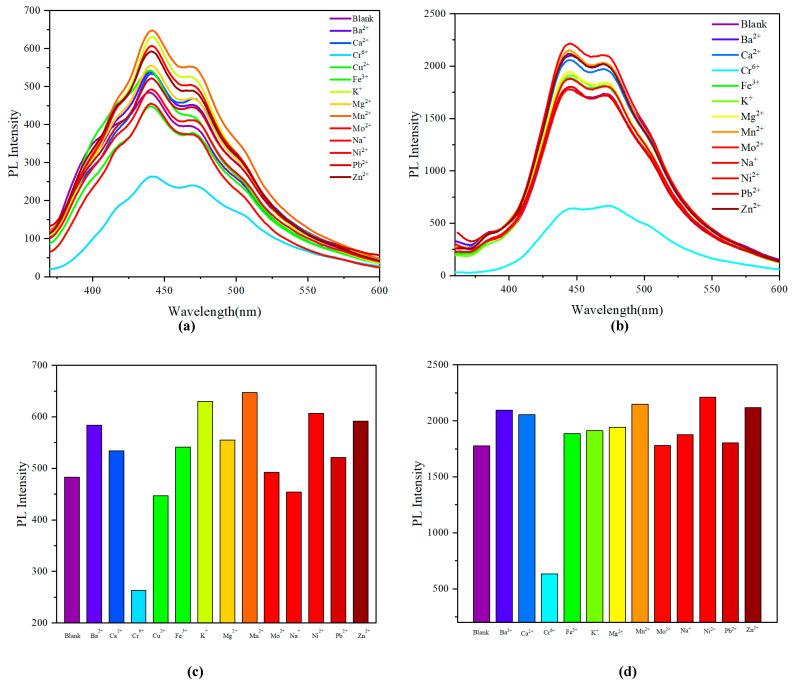
(**a**,**b**) Fluorescence emission profile of polymers **2A** and **3A** (0.05 mg/mL) in the presence of various metals ion solvents of Ba^2+^, Ca^2+^, Cr^6+^, Cu^2+^, Fe^3+^, K^+^, Mg^2+^, Mn^2+^, Mo^2+^, Na^+^, Ni^2+^, Pb^2+^, and Zn^2+^ 5 µL in PBS buffer (20 mM pH 7.4) solution (THF). Excitation wavelength: 331 nm. (**c**,**d**) Fluorescence response of 0.05 mg/mL polymer **2A** and **3A** to various metal ions. Excitation at 331 nm.

Even though several new multilayer 3D chirality structures have been developed recently, their practical applications are still extremely limited. Following our study of the AIE and AIEE phenomenon, we initiated our investigation into the potential for identifying distinct metal ions within a given solvent system. The selectivity and competitive experiments were performed in THF. Various metal cations (Ba^2+^, Ca^2+^, Cr^6+^, Cu^2+^, Fe^3+^, K^+^, Mg^2+^, Mn^2+^, Mo^2+^, Na^+^, Pb^2+^ and Zn^2+^) were added to the solution of **2A** and **3A** under the same condition separately, and the corresponding fluorescence intensities were recorded. As shown in Figure 6a,b, the new obvious quenched emission peaked around 450 nm was observed. The histogram of fluorescence intensities at 450 nm was established for each system, as shown in Figure 6c,d. The presence of other metal cations could hardly interfere a lot with the fluorescence intensities of polymer **2A** and **3A**, indicating that polymer **2A** and **3A** had a strong anti-interference ability, and Cr^6+^ could be effectively recognized by polymer **2A** and **3A**.

**Figure 7:**
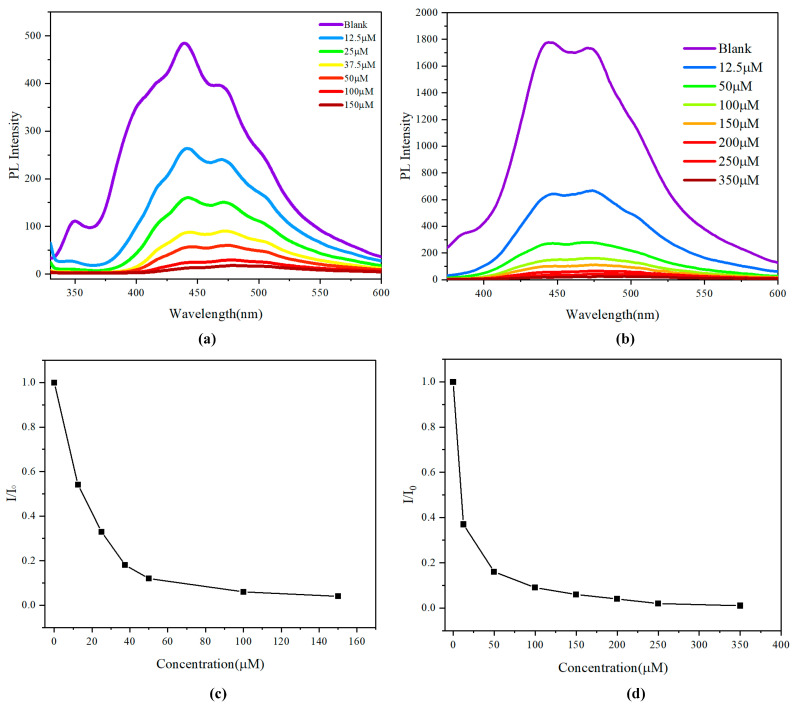
(**a**,**b**). Concentration-dependent fluorescence spectra of **2A** and **3A** (0.05 mg/mL) on the addition of various amounts of Cr^6+^ (0–350 µM) in PBS buffer (20 mM pH 7.4) solution (THF). The excitation wavelength was 331 nm. (**c**,**d**) Stern–Volmer plots of I/I_0_ vs. Cr^6+^ concentration for **2A** and **3A**.

According to the above analysis, we found a phenomenon whereby chiral multilayered 3D polymers exhibit enhanced sensitivity in detecting Cr^6^⁺ ions compared to their achiral counterpart. The alkyne-based polymer exhibits enhanced solubility and stability in THF, which is critical for maintaining the structural integrity of the polymer during the sensing process. In contrast, exposure to water could lead to hydrolysis or other degradation pathways that would compromise the polymer’s functionality. The sensing mechanism relies on specific interactions between the polymer and Cr^6+^ ions, optimized in a non-polar or mildly polar solvent like THF. The presence of water, a highly polar solvent, could alter these interactions, potentially leading to reduced sensitivity and selectivity for Cr^6+^ detection. Aqueous environments could introduce additional ions and molecules that could interfere with detecting Cr^6+^, further complicating the sensing process and leading to unreliable results (Figure 7).

Chiral materials often possess unique spatial arrangements that allow for selective interactions with specific molecules. The presence of chiral centers within the multilayered polymer can facilitate preferential binding or recognition of Cr^6^⁺ ions, potentially enhancing the interaction strength through stereospecific effects, and this selective binding can lead to a more pronounced response in the presence of the target ion. On the other hand, Chiral polymers could exhibit distinct electronic properties due to their asymmetric structures. The arrangement of electronic states in chiral materials can lead to increased sensitivity through mechanisms such as charge transfer or enhanced conductivity upon interaction with Cr^6^⁺ ions, which can result in a more significant change in the optical or electronic response of the chiral multilayered polymer when exposed to the target ion. According to structural features and surface area, the multilayered architecture of the chiral polymer could provide a greater surface area for interaction with Cr^6^⁺ ions compared to an achiral multilayered structure; this increased surface area can enhance the availability of binding sites, allowing for a higher concentration of ions to interact with the polymer matrix, thereby improving detection sensitivity. In interlayer interactions, the arrangement of layers could facilitate interlayer interactions that are not present in achiral systems, and these interactions can promote cooperative binding effects, where the binding of one Cr^6^⁺ ion enhances the binding affinity of subsequent ions, leading to an amplified overall response. The ability of an alkyne-based multilayered 3D polymer to detect Cr^6^⁺ ions indicates a promising advancement in sensor technology, with implications for environmental monitoring, industrial safety, and public health; this capability highlights the importance of innovative materials in addressing critical challenges associated with toxic pollutants.

### 3.3. CD Spectroscopy

Chirality refers to the geometric property of a molecule that makes it non-superimposable on its mirror image. Chiral molecules exist in two enantiomeric forms (e.g., left-handed and right-handed), which can exhibit different optical activities. CD spectroscopy measures the differential absorption of left-handed and right-handed circularly polarized light by chiral molecules. This differential absorption leads to a characteristic CD spectrum, which provides information about the molecular structure and conformation. CD spectroscopy plays a crucial role in measuring chirality by providing valuable structural information, enabling quantitative analysis, and facilitating applications across multiple scientific disciplines. Its ability to differentiate between enantiomers and characterize their behavior in various environments makes it an indispensable tool in the study of chiral molecules.

**Figure 8:**
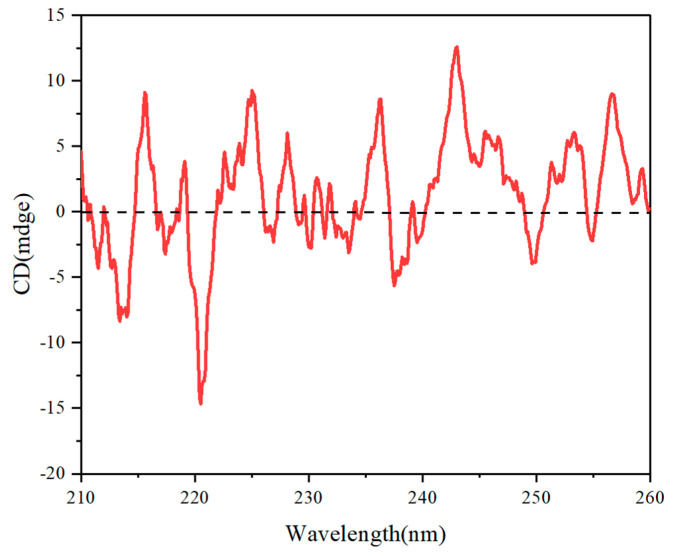
CD Spectroscopy of polymer **3A** in THF. (c = 0.2 mg/mL).

With CD spectroscopies, the optical activity of chiral polymers **3A** in THF was further investigated. Because of the aromatic rings’ π-π* transition, there was only one optical absorption that showed up between 210 and 260 nm. Polymer **3A** had evident negative cotton effects in the following ranges: 210–215 nm, 218–220 nm, 223–225 nm, and 235–250 nm. On the other hand, it demonstrated positive cotton effects in the following ranges: 213–217 nm, 220–236 nm, 233–236 nm, and 238–245 nm. These results are shown in Figure 8. The obvious cotton effect in CD spectra is significant as it indicates the presence of chirality, provides insights into molecular conformation and interactions, and can help differentiate between enantiomers.

### 3.4. Analysis of Particle Size by SEM and DLS

**Figure 9:**
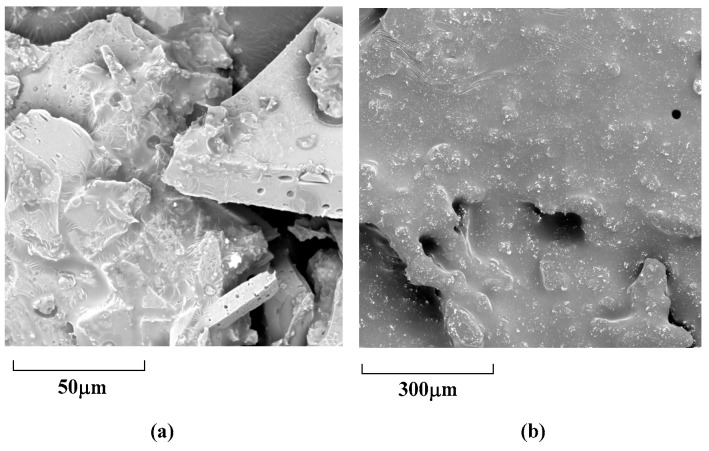
SEM images of polymers **2A** and **3A** (**a**,**b**).

Scanning electron microscopy (SEM) is primarily used for imaging and analyzing the surface morphology and topography of materials at high resolutions. While SEM itself is not a direct method for measuring chirality, it plays an important role in the study of chiral materials and their applications. The chiral polymers **2A** and **3A** conducted morphological investigations with scanning electron microscopy (SEM). All of the polymer samples were coated with a tiny layer of gold to improve their conductivity and lower their signal-to-noise ratio (Figure 9).

According to the picture of achiral and chiral polymers **2A** and **3A** that were tested, there is only a small amount of a single particle left and no crystalline structure in the entire polymer system. In the situation where there is a high degree of polymerization, the majority of the particles have the large shape of a stone block. This demonstrates that the polymerization and aggregation process is going fairly well and that an irregular packing process occurred during the process of polymerization, which caused there to be a comparatively small amount of monomer and oligomer particles. Since the picture does not exhibit any linear components, its extension is extremely constrained.

**Figure 10:**
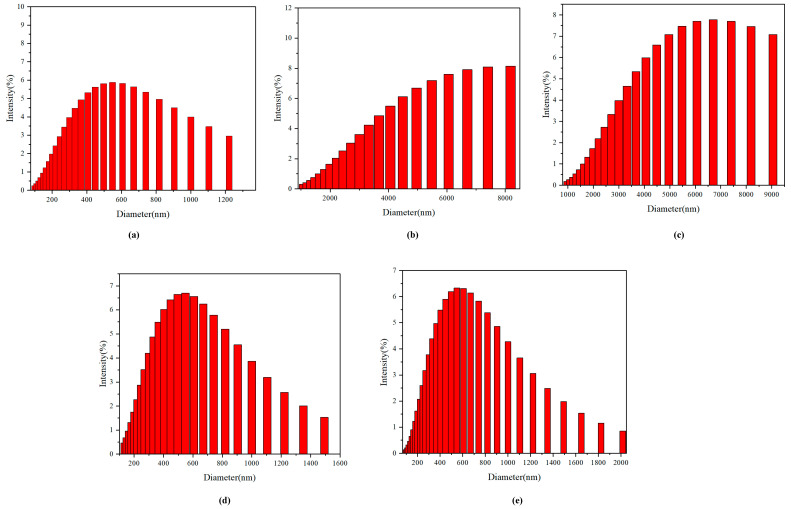
(**a**–**c**) DLS particle size distribution curves of **1A**, **2A**, and **3A** in THF. (**d**,**e**) DLS particle size distribution curves of **2A** and **3A** with Cr^6+^ ion.

Scanning electron microscopy reflects a stone-like surface, which leads us to believe that the monomer **1A** and synthetic polymers **2A** and **3A** have huge particle sizes. We study the phenomenon through dynamic light scattering to detect improved aggregation and quench compared to formerly published multilayered 3D polymers. Dynamic light scattering (DLS) experiments reveal that the largest diameter of aggregates for monomer **1A** is 548 nm (Figure 10a). Following polymerization by monomer **1**, the diameter of particle sizes for **2A** and **3A** increased by nearly ten times (Figure 10b,c); the largest diameters for **2A** are 7504 nm, and the largest diameter for **3A** is 6700 nm. When monomers are polymerized, they undergo a series of chemical reactions that lead to the formation of larger polymer chains. In the case of multilayered structures, these chains can interact with one another through various intermolecular forces, such as van der Waals forces, hydrogen bonding, and hydrophobic interactions.

Since the addition of Cr^6+^ ions causes the quench phenomena, which is shown in photoluminescence (Figure 8), we assume that the Cr^6+^ ions could hinder the aggregation process when multilayered 3D polymer is packed as an assembly. We maintained the Cr^6+^ ion concentration of 12 µm in polymers **2A** and **3A** solution independently. We discovered that the largest diameter polymer particles were 606 nm and 670 nm, which were approximately 10% of particles before the addition of the Cr^6+^ ion (Figure 10d,e). This significant reduction in particle size implies that the presence of Cr^6+^ ions could hinder the aggregation process, likely due to the introduction of steric hindrance or electrostatic repulsion among the polymer chains. The Cr^6+^ ions could disrupt the intermolecular forces that typically facilitate particle growth, leading to the formation of smaller, more stable assemblies. This phenomenon is critical as it not only affects the physical dimensions of the polymer particles but also has implications for their functional properties, such as surface area, reactivity, and potential applications in fields like drug delivery and catalysis.

## 4. Conclusions

In conclusion, this study investigates the synthesis and characterization of alkyne-based multilayered three-dimensional (3D) polymers exhibiting aggregation-induced emission (AIE). Their AIE properties and ability to selectively detect chromium (VI) ions (Cr^6^⁺) position these polymers as promising fluorescent probes for environmental sensing. The pronounced cotton effect in the circular dichroism (CD) spectra confirms their chiral nature, enhancing their applicability in chiral studies. Morphological analysis via scanning electron microscopy (SEM) and dynamic light scattering (DLS) reveals a unique rock-like surface morphology essential for target–analyte interaction. These findings highlight the multifunctional potential of these polymers in fluorescence-based sensing and materials science. Looking ahead, future research will focus on exploring multi-component reactions [23,24,25,26] and advancements in nanotechnology applications [27,28,29,30]. This approach promises to further enhance the functionality and versatility of these polymers, potentially leading to groundbreaking developments in various scientific and industrial fields.

## Data Availability

Data are contained within the article and Supplementary Materials.

## Supplementary Materials

The following supporting information can be downloaded at: https://www.mdpi.com/article/10.3390/molecules29235641/s1, Figure S1–S6: NMR and GPC spectrum.

## References

[1] Wu G., Liu Y., Yang Z., Katakam N., Rouh H., Ahmed S., Unruh D., Surowiec K., Li G. (2019). Multilayer *3D* Chirality and Its Synthetic Assembly. Research.

[2] Wu G., Liu Y., Yang Z., Jiang T., Katakam N., Rouh H., Ma L., Tang Y., Ahmed S., Rahman A.U. (2020). Enantioselective Assembly of Multi-Layer *3D* Chirality. Natl. Sci. Rev..

[3] Zhang J., Kürti L. (2021). Multi-Layer 3D Chirality: Its Enantioselective Synthesis and Aggregation-Induced Emission. Natl. Sci. Rev..

[4] Liu Y., Wu G., Yang Z., Rouh H., Katakam N., Ahmed S., Unruh D., Cui Z., Lischka H., Li G. (2020). Multi-Layer 3D Chirality: New Synthesis, AIE and Computational Studies. Sci. China Chem..

[5] Chen H., Wu W., Zhu J., Yang S.A., Zhang L. (2021). Propagating Chiral Phonons in Three-Dimensional Materials. Nano Lett..

[6] Li G., Liu Y., Rouh H., Tang Y., Wu G., Yuan Q., Zhang S., Wang J.-Y., Jin S., Xu T. (2023). Enantio- and Diastereoselective Assembly of Multi-Layer Folding Chiral Targets via Asymmetric Catalytic Single C–C Bond Formation. Synlett.

[7] Zeraati M., Langley D.B., Schofield P., Moye A.L., Rouet R., Hughes W.E., Bryan T.M., Dinger M.E., Christ D. (2018). I-Motif DNA Structures Are Formed in the Nuclei of Human Cells. Nat. Chem..

[8] Fan Z., Iqbal H., Ni J., Khan N.U., Irshad S., Razzaq A., Alfaifi M.Y., Elbehairi S.E.I., Shati A.A., Zhou J. (2024). Rationalized Landscape on Protein-Based Cancer Nanomedicine: Recent Progress and Challenges. Int. J. Pharm. X.

[9] Dao F.-Y., Lv H., Fullwood M.J., Lin H. (2022). Accurate Identification of DNA Replication Origin by Fusing Epigenomics and Chromatin Interaction Information. Research.

[10] Xia Q., Liu R., Chen X., Chen Z., Zhu J.-J. (2023). In Vivo Voltammetric Imaging of Metal Nanoparticle-Catalyzed Single-Cell Electron Transfer by Fermi Level-Responsive Graphene. Research.

[11] Wu G., Liu Y., Yang Z., Ma L., Tang Y., Zhao X., Rouh H., Zheng Q., Zhou P., Wang J.-Y. (2021). Triple-Columned and Multiple-Layered 3D Polymers: Design, Synthesis, Aggregation-Induced Emission (AIE), and Computational Study. Research.

[12] Chang C.H., Hsieh C.H., Huang J.C., Wang C., Liao Y.C., Hsueh C.H., Du X.H., Wang Z.K., Wang X. (2016). Designing a Stronger Interface Through Graded Structures in Amorphous/Nanocrystalline ZrCu/Cu Multilayered Films. Nanotechnology.

[13] Legrand W., Chauleau J.-Y., Maccariello D., Reyren N., Collin S., Bouzehouane K., Jaouen N., Cros V., Fert A. (2018). Hybrid Chiral Domain Walls and Skyrmions in Magnetic Multilayers. Sci. Adv..

[14] Zhang S., Yuan Q., Li G. (2024). New Multiple-Layered 3D Polymers Showing Aggregation-Induced Emission and Polarization. RSC Adv..

[15] Ai C., Zhang Y., Zhang S., Yan S. (2024). Thiophene-Based Multi-Layered 3D Chiral Polymer and Oligomer Containing Aggregate-Induced Emission and Polarization. ChemistrySelect.

[16] Dong G., Pan Z., Han B., Tao Y., Chen X., Luo G.-G., Sun P., Sun C., Sun D. (2023). Multi-Layer 3D Chirality and Double-Helical Assembly in a Copper Nanocluster with a Triple-Helical Cu15 Core. Angew. Chem. Int. Ed Engl..

[17] Sonogashira K., Tohda Y., Hagihara N. (1975). A Convenient Synthesis of Acetylenes: Catalytic Substitutions of Acetylenic Hydrogen with Bromoalkenes, Iodoarenes and Bromopyridines. Tetrahedron Lett..

[18] Takahashi S., Kuroyama Y., Sonogashira K., Hagihara N. (1980). A Convenient Synthesis of Ethynylarenes and Diethynylarenes. Synthesis.

[19] Liu Y., Lam J.W.Y., Tang B.Z. (2015). Conjugated Polymers Developed from Alkynes. Natl. Sci. Rev..

[20] Wang H., Li Q., Alam P., Bai H., Bhalla V., Bryce M.R., Cao M., Chen C., Chen S., Chen X. (2023). Aggregation-Induced Emission (AIE), Life and Health. ACS Nano.

[21] Mei J., Leung N.L.C., Kwok R.T.K., Lam J.W.Y., Tang B.Z. (2015). Aggregation-Induced Emission: Together We Shine, United We Soar!. Chem. Rev..

[22] Luo J., Xie Z., Lam J.W.Y., Cheng L., Tang B.Z., Chen H., Qiu C., Kwok H.S., Zhan X., Liu Y. (2001). Aggregation-Induced Emission of 1-Methyl-1,2,3,4,5-Pentaphenylsilole. Chem. Commun..

[23] Ma X., Zhi S., Zhang W. (2021). Recent Developments on Five-Component Reactions. Molecules.

[24] Zhou J., Zhou Q., Wan J.-P. (2024). Recent Advances in the Multicomponent Synthesis of Pyrazoles. Org. Biomol. Chem..

[25] Reguera L., Rivera D.G. (2019). Multicomponent Reaction Toolbox for Peptide Macrocyclization and Stapling. Chem. Rev..

[26] Yang G., Sun L., Zhang Q. (2024). Multicomponent Chiral Plasmonic Hybrid Nanomaterials: Recent Advances in Synthesis and Applications. Nanoscale Adv..

[27] Zhang J., Shen Y., Jin N., Zhao X., Li H., Ji N., Li Y., Zha B., Li L., Yao X. (2022). Chemo-Biocascade Reactions Enabled by Metal-Organic Framework Micro-Nanoreactor. Research.

[28] Huang Y., Ouyang W., Lai Z., Qiu G., Bu Z., Zhu X., Wang Q., Yu Y., Liu J. (2024). Nanotechnology-Enabled Sonodynamic Therapy Against Malignant Tumors. Nanoscale Adv..

[29] He L., Zhang W., Liu J., Pan Y., Li S., Xie Y. (2024). Applications of Nanotechnology in Orthodontics: A Comprehensive Review of Tooth Movement, Antibacterial Properties, Friction Reduction, and Corrosion Resistance. Biomed. Eng. Online.

[30] Ramezani G., Stiharu I., van de Ven T.G.M., Nerguizian V. (2024). Advancements in Hybrid Cellulose-Based Films: Innovations and Applications in 2D Nano-Delivery Systems. J. Funct. Biomater..

