# Supplementary material for: Exploration of Alkyne-Based Multilayered 3D Polymers and Oligomers: Subtle Aggregation-Induced Emission, Chromium(VI) Ion Detection, and Chiral Properties Characterization
Source: Molecules. 2024 Nov 28;29(23):5641. doi: 10.3390/molecules29235641 (PMC11643666; doi:10.3390/molecules29235641)
Supplement: Supplementary file 1 [file molecules-29-05641-s001.zip › molecules-3319177-supplementary.pdf]

Exploration of Alkyne-Based Multilayered 3D Polymers and Oligomers: Subtle Aggregation-Induced Emission, Chromium(VI) Ion Detection, and Chiral Properties Characterization

Sai Zhang, [1]* Qingzheng Xu,[2] Xiuyuan Qin,[3] Jialin Mao,[5] Yue Zhang,[1,5] and Guigen Li,[4]*

[1]School of Pharmacy, Continuous Flow Engineering Laboratory of National Petroleum and Chemical Industry, Changzhou University, Changzhou, Jiangsu Province, 213164, China

[2] School of Chemistry and Chemical Engineering, Nanjing University, Nanjing, Jiangsu Province, 210093, China

[3] School of Life and Science, Nanjing Normal University, Nanjing, Jiangsu Province, 210046, China

[4] Department of Chemistry and Biochemistry, Texas Tech University, Lubbock, Texas, 79409-1061, USA

[5] School of Environmental Science and Engineering, Changzhou University, Changzhou, Jiangsu Province, 213164, China.

General Information

All processes were magnetically stirred in oven-dried glassware with anhydrous solvents under Ar. Syringes, stainless steel or polyethylene cannulas, rubber septa, or a weak Ar counter-flow adding solvents, liquids, and solutions. Ice/water (0 °C) or dry ice/acetone (-78 °C) cooling baths were created in Dewar vessels. High-temperature processes used heated oil baths. Rotavapors at 40-65 °C eliminated solvents. All yields are separate chromatographic and NMR yields.

Without additional purification, all commercially accessible compounds were utilized as received. Without further purification, solvents such CH_3OH, toluene, EA, ether, DCM, dioxane, and acetone were employed. An innovative technology solvent system delivers THF and DCM.

On 400 MHz and 500 MHz instruments with TMS as an internal standard, the ^1H and ^{13}C NMR spectra were captured. The residual solvent signal (= 7.26 for $CDCl^3$) was utilized to reference the 1H NMR spectra. The signal of the solvents was employed in the 13C NMR spectra (= 7.16 for $CDCl^3$ and). Chemical shifts () about TMS were reported in ppm. Chemical shift, multiplicity (singlet, doublet, triplet, multiplet), coupling constant (J, Hz), and integration are used to describe the data. The TOSOH EcoSEC HLC-8420 GPC, which has a dual-flow refractive index detector, was used to collect GPC data. Along with the RI detector, a UV detector is also provided for UV-visible polymers. The range of the installed columns is 500-107 Da. Samples were conducted at a flow rate of 0.7 mL/min for 25 minutes. Our investigations used polystyrene (PS) standards for calibration.

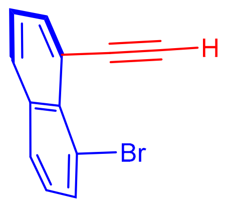

1A

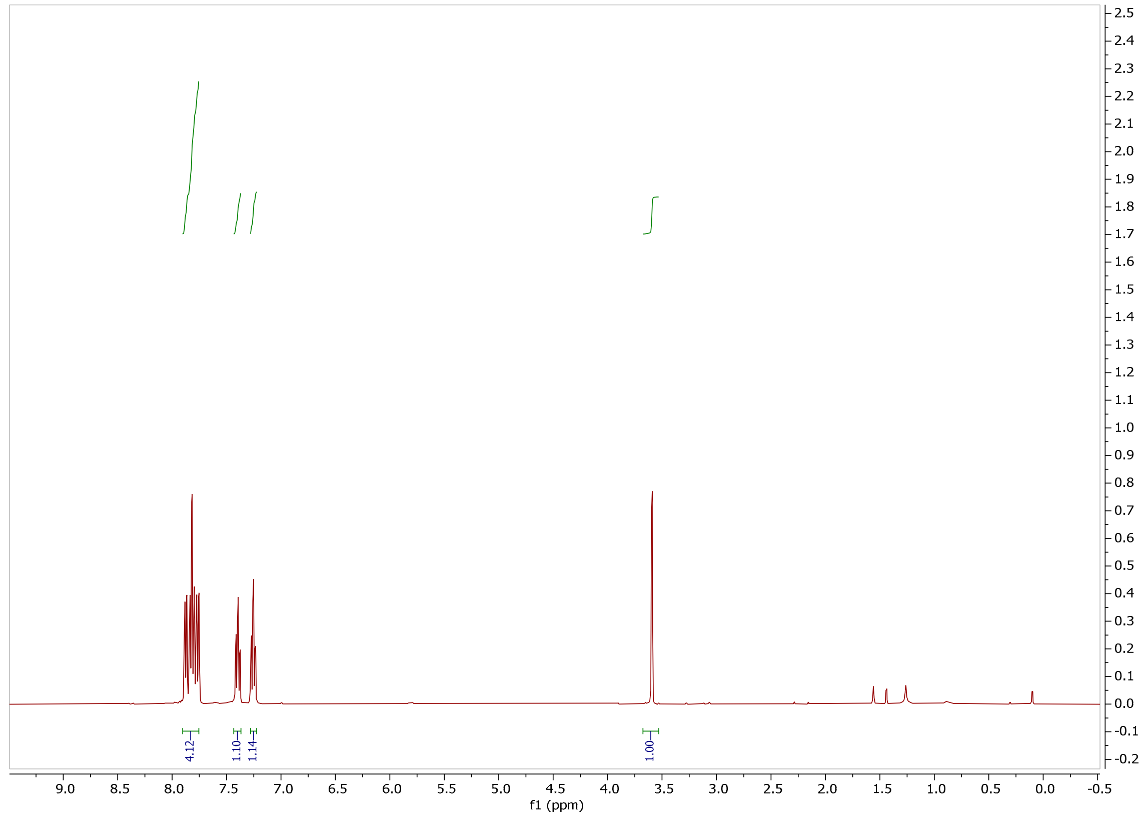

Figure S1. ¹H NMR spectrum of **1A**

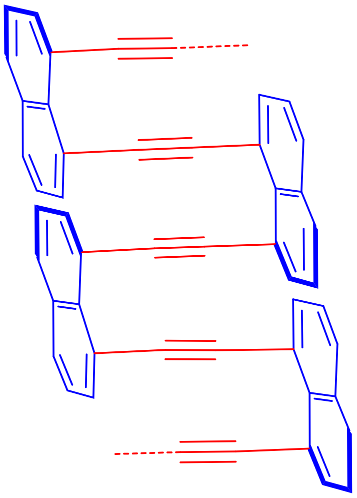

2A

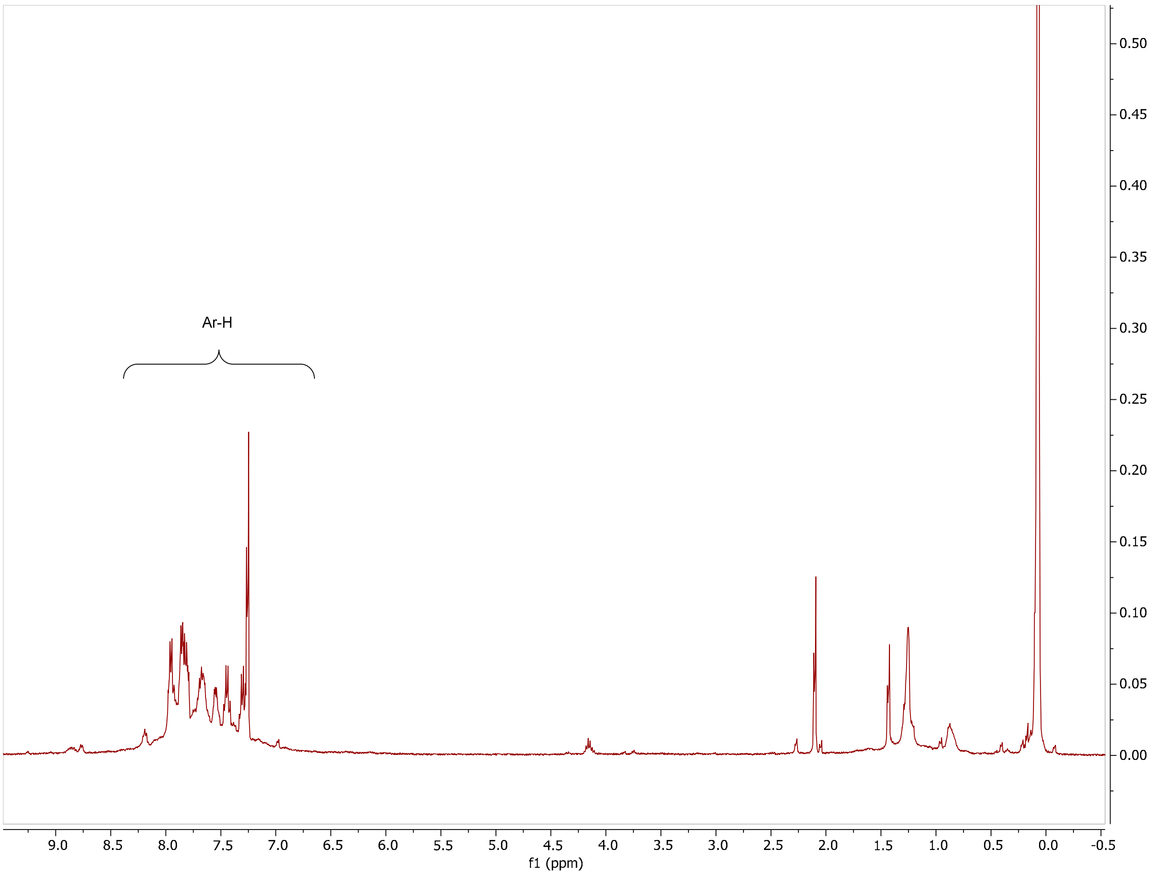

Figure S2. ^{1}H NMR spectrum of **2A**

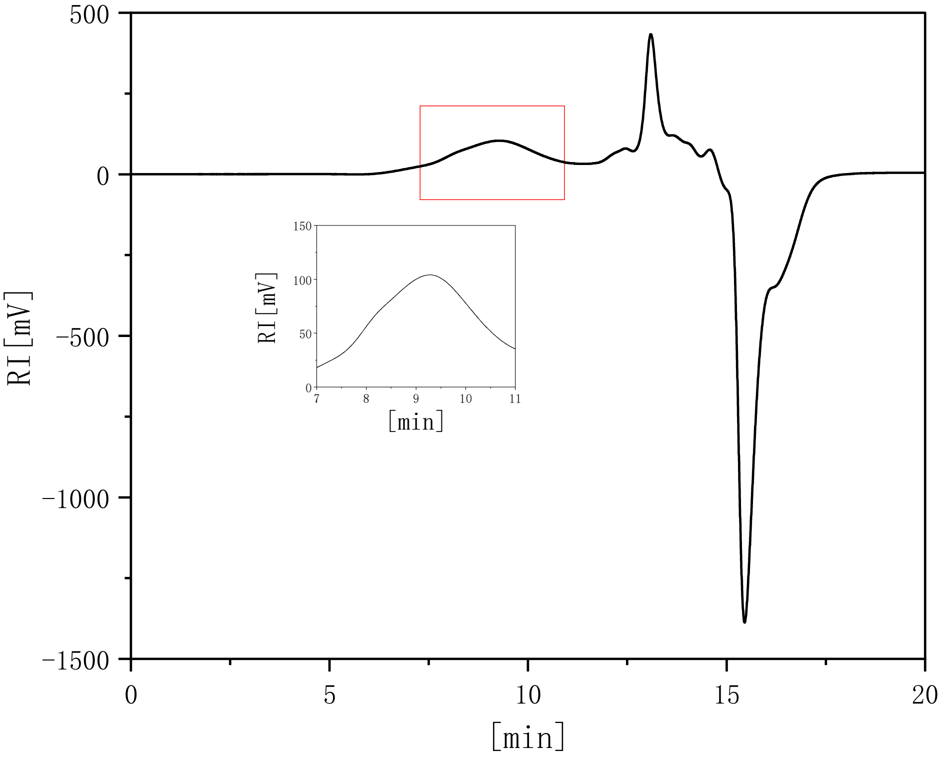

Figure S3.GPC data of **2A**

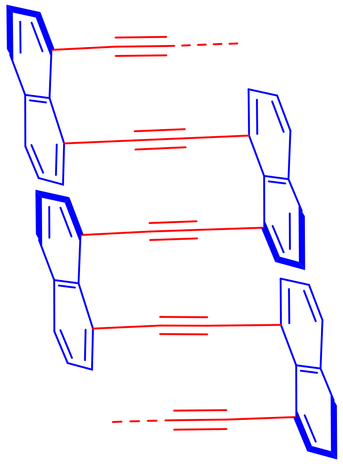

3A

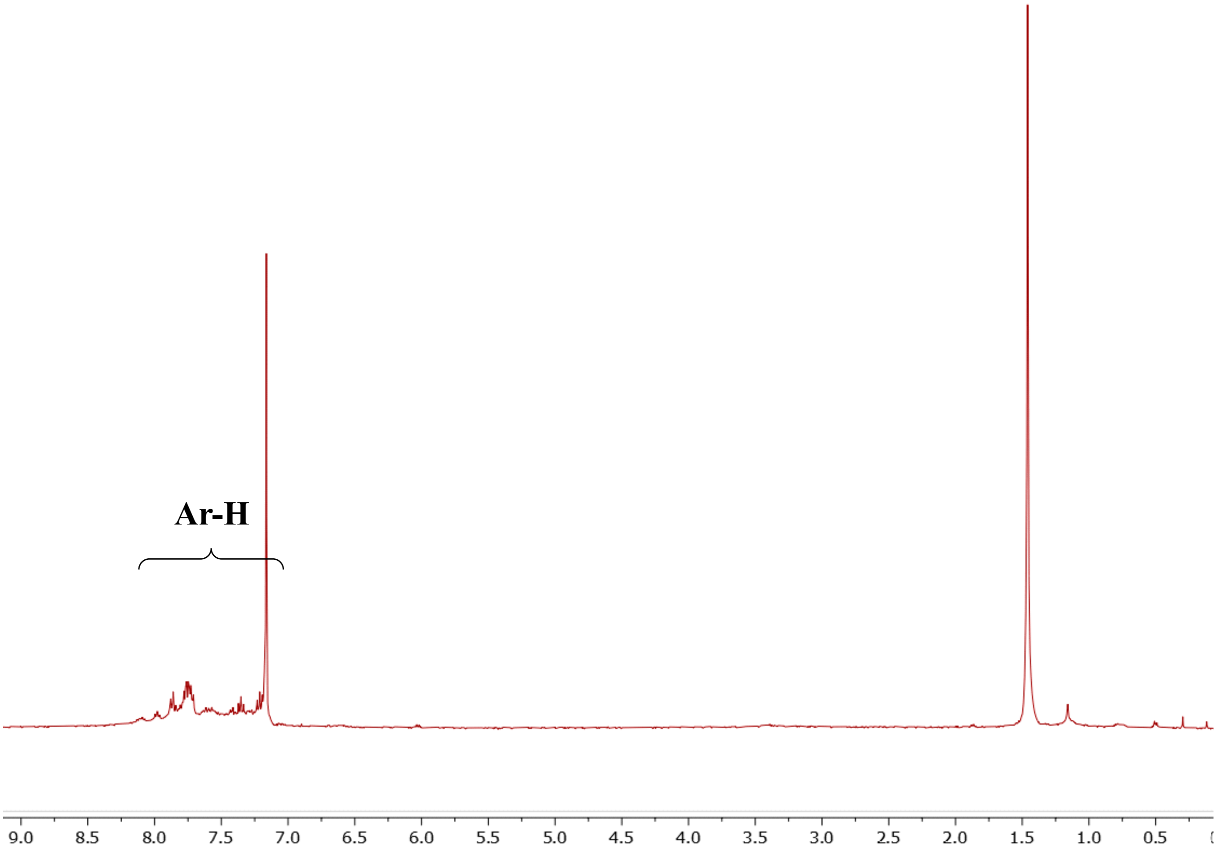

Figure S4. ^{1}H NMR spectrum of **3A**

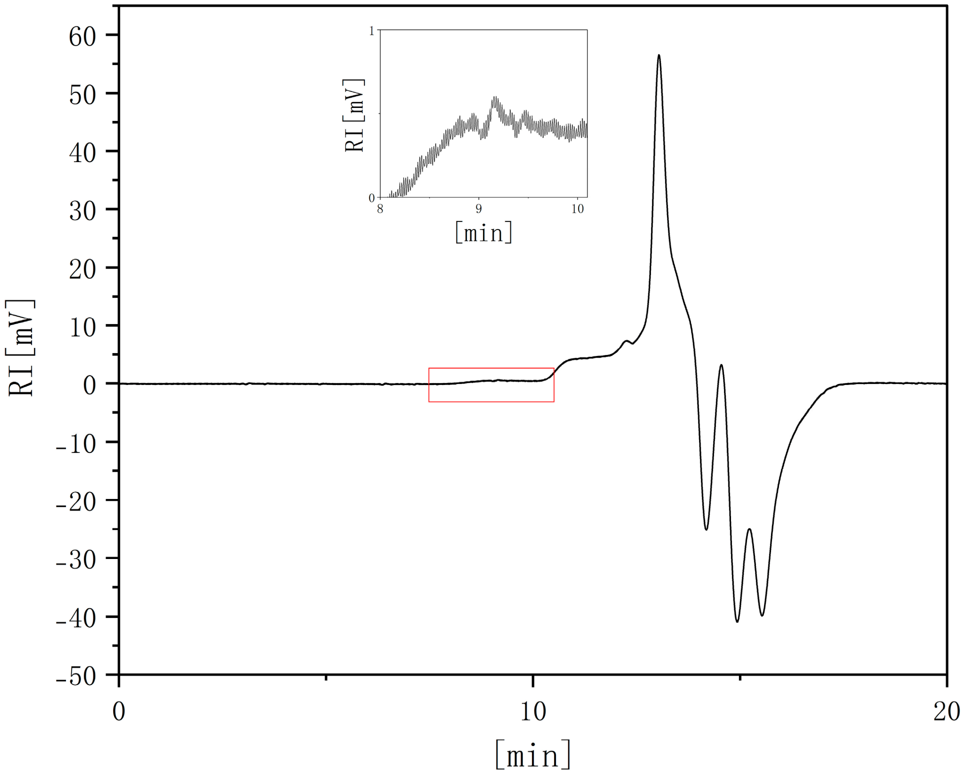

Figure S5. GPC spectrum of 3A

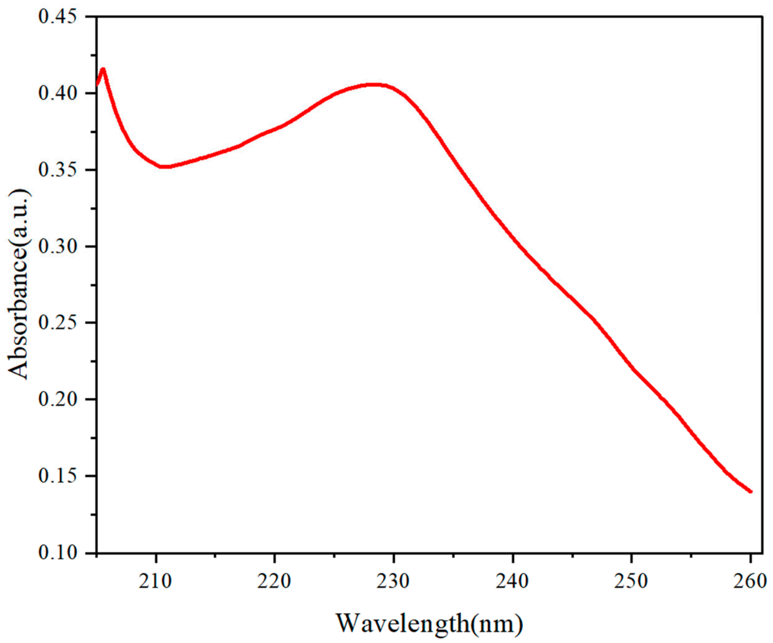

Figure S6. UV range of CD Spectra
